# Both piston-like and rotational motions are present in bacterial chemoreceptor signaling

**DOI:** 10.1038/srep08640

**Published:** 2015-03-02

**Authors:** Daqi Yu, Xiaomin Ma, Yuhai Tu, Luhua Lai

**Affiliations:** 1 BNLMS, State Key Laboratory for Structural Chemistry of Unstable and Stable Species, College of Chemistry and Molecular Engineering, Peking University, Beijing. 100871, China; 2 Center for Quantitative Biology, Academy for Advanced Interdisciplinary Studies, Peking University, Beijing. 100871, China; 3 IBM T. J. Watson Research Center, Yorktown Heights, New York 10598, USA; 4 Peking-Tsinghua Center for Life Sciences, Peking University, Beijing. 100871, China

## Abstract

Bacterial chemotaxis signaling is triggered by binding of chemo-effectors to the membrane-bound chemoreceptor dimers. Though much is known about the structure of the chemoreceptors, details of the receptor dynamics and their effects on signaling are still unclear. Here, by using molecular dynamics simulations and principle component analysis, we study the dynamics of the periplasmic domain of aspartate chemoreceptor Tar dimer and its conformational changes when binding to different ligands (attractant, antagonist, and two attractant molecules). We found two dominant components (modes) in the receptor dynamics: a relative rotation of the two Tar monomers and a piston-like up-and-down sliding movement of the α4 helix. These two modes are highly correlated. Binding of one attractant molecule to the Tar dimer induced both significant piston-like downward movements of the α4 helix and strong relative rotations of the two Tar monomers, while binding of an antagonist or the symmetric binding of two attractant molecules to a Tar dimer suppresses both modes. The anti-symmetric effects of the relative rotation mode also explained the negative cooperativity between the two binding pockets. Our results suggest a mechanism of coupled rotation and piston-like motion for bacterial chemoreceptor signaling.

Bacterial chemotaxis system is an important member of the general two-component regulatory systems that enable bacteria to sense and to react to changes in their environmental conditions^1^,^2^. The transmembrane methyl-accepting chemotaxis proteins (MCP) are primarily responsible for sensing different chemical stimuli. The MCPs also play important roles in signal processing, such as signal amplification and adaptation^2^. There are many kinds of MCPs in bacteria. The aspartate (Asp) chemoreceptor Tar and the serine (Ser) chemoreceptor Tsr are the major chemoreceptors in *Escherichia coli* (*E. coli*). Most of our study here are focused on Tar.

Two Tar monomers form a stable homodimer^3^, which provides two symmetric binding pockets at the interface of the two monomers^4^. Negative cooperativities were found between the two binding pockets^5^. Several crystal structures for Tar have been solved under different crystallization conditions^4^,^6^,^7^,^8^,^9^,^10^,^11^. By comparing the crystal structures, it has been proposed that the transmembrane signaling was triggered by a relative piston-like downward sliding of the α4 helix in the periplasmic domain after attractant binding^1^,^12^,^13^,^14^. The piston-like model is supported by several experiments^15^,^16^,^17^. Molecular dynamics (MD) simulations of transmembrane helices with different sequences also support the piston-like model^18^. In addition, recent MD simulations^19^ showed that restrained piston-like downward sliding of the α4 helix in the periplasmic domain can propagate the signal by affecting the transmembrane and the (cytoplasmic) HAMP domains of the receptor. However, other kinds of motions such as inter-monomer rotations have also been reported^9^,^10^,^11^,^20^,^21^.

Besides Asp, several other amino acids also act as attractants for Tar, albeit with weaker sensitivities^22^,^23^. Metal ions, such as Ni^2+^ and Co^2+^, act as repellents of *E. coli* though Tar^24^, though the molecular mechanism is unclear. In addition to attractants and repellents, molecules that bind to chemoreceptors directly^25^ or through binding proteins^26^ without causing any chemotactic responses also exist^25^. Recently, by using an integrated computational and experimental approach, we have discovered several previously unknown *E. coli* Tar chemoeffectors^25^. Aside from the novel attractant molecules, such as, α-amino-3-hydroxy-5-methyl-4-isoxazolepropionic acid, guanidinosuccinic acid, we also found two antagonists, *cis*-1, 2-cyclohexane-dicarboxylic acid (CHDCA) and phthalic acid (PA), which directly bind to Tar without causing any chemotactic responses. We found that subtle changes in the ligand structure could have large effects on its function. In particular, an amino analog of CHDCA, *cis*-(2R, 3S)-2, 3-piperidine dicarboxylic acid (*cis*-PDA), turns out to be an attractant. Given that attractants and antagonists of Tar bind to the same binding pocket as the cognate attractant (Asp), it raises the interesting question of how they induce different chemotactic responses.

Here, to understand the molecular mechanisms of attractant and antagonist functions, we performed molecular simulations for the periplasmic domain (residues from 36 to 181) of *E. coli* Tar dimer by itself and when bound with different attractant and antagonist molecules. Specifically, MD simulations were carried out for *E. coli* Tar dimer in 5 different cases: the apo system without any ligand, holo systems with a bound Asp, a bound CHDCA, a bound *cis*-PDA, and two bound Asps. The apo structure has a broad conformation distribution, and ligand binding in general tightens the structure. Principal component analysis (PCA) was performed and two dominant intrinsic principal components (modes) were found. The first mode corresponds to the inter-monomer rotation and the second mode corresponds to the piston-like α4 helix sliding motion. The two modes were found to be highly correlated. Binging to a single attractant (1 Asp or 1 *cis*-PDA) induced both significant inter-monomer rotations and piston-like downward movements. Binding to one antagonist CHDCA or two Asp molecules suppressed both modes in the Tar dimer. Analysis of the binding free energy contributions for Tar binding to attractant or antagonist molecules support the functional relevance of the hydrogen bonds between the ligands and the α4 helix of Tar.

## Results

**Figure 1:**
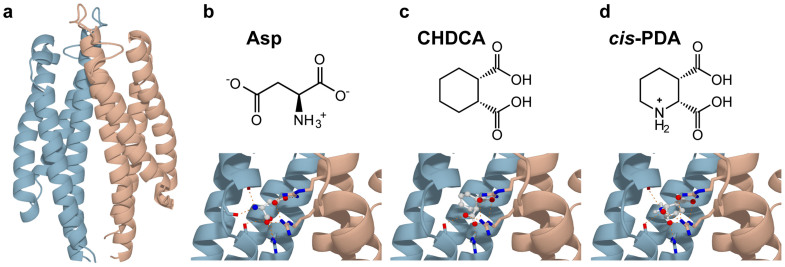
Starting structures for the simulation systems. (a) The initial structure for the apo system. The holo systems are bound with (b) 1 Asp, (c) 1 CHDCA, (d) 1 *cis*-PDA, and 2 Asps where each of the two pockets was occupied by an Asp, respectively. Two monomers of Tar dimer were shown in light blue and light red, respectively. The hydrogen bonds between the ligands and the binding pockets of Tar were labeled as orange lines. The ligands were shown in ball-and-stick model.

In this paper, we studied five different systems: 1) the apo system (*E. coli* Tar periplasmic domain, with residues 36–181) without any ligand, 2) Tar dimer binding with one Asp, 3) Tar dimer binding with one CHDCA, 4) Tar dimer binding with one *cis*-PDA, 5) Tar dimer binding with two Asps. Details of the systems are given in Supplementary Table S1. The initial structures for the simulation systems were shown in Figure 1. During the simulations, the ligands were confirmed to reside in the binding pockets as the center of mass distances between the ligands and the protein were shown to have little drifts and limited fluctuations in all simulations (see Supplementary Fig. S1).

### A broad conformation distribution for the apo system

In MD simulations, all the eight helices (four for each monomer with the following residues: 44–75, 89–113, 118–144, 146–175, respectively) in the periplasmic domain show smaller residue fluctuations than the loop regions (see Supplementary Fig. S2). To explore conformational changes, we fitted and calculated the helix C_α_ root mean square deviation (RMSD) and distance root mean square (DRMS)^27^ for each pair of trajectory snapshots.

**Figure 2:**
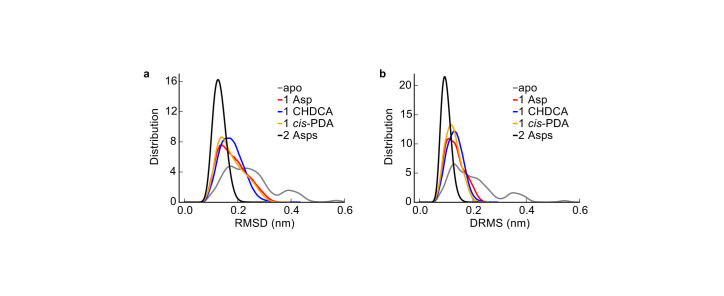
Distributions of the structure differences. (a) RMSD distribution and (b) DRMS distribution of the apo and holo systems. The distributions were determined from the structures taken from the MD trajectories every 100 ps.

The apo system was found to be more flexible with much broader RMSD and DRMS distributions than the holo systems (see Fig. 2). In this sense, ligand binding stabilized structures of Tar periplasmic domain. This result is compatible with the frozen dynamic dimer model for periplasmic domain^21^. Note that this ligand binding induced tightening effect may not be valid in cytoplasmic regions of the receptor away from its periplasmic binding pocket. In fact, there are reports of both loosened and tightened dynamics in different regions of the cytoplasmic domain in bacterial chemoreceptors upon attractant binding^28^,^29^,^30^.

Based on the DRMS distributions (Fig. 2b), the structures in the trajectories were clustered with a DRMS cutoff value of 0.2 nm. Three clusters were derived for the apo system, with the biggest cluster containing 85% of all structures in the simulation. In contrast, there was only one cluster for each holo system.

### The two intrinsic functional modes in the apo system

**Figure 3:**
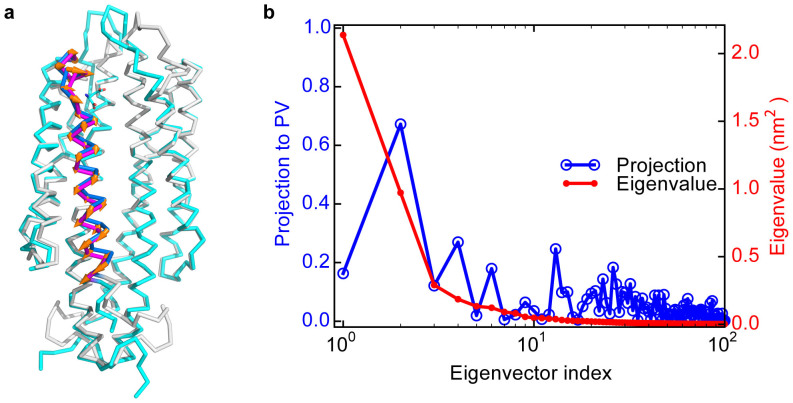
Illustrations of PV and the principal components. (a) Illustration of the piston vector (PV). The apo structure is blue and gray colored. The holo structure is shown in cran and magenta colors. PV is the normalized C_α_ displacements from blue to magenta helix (parallel to the orange arrows). (b) The projections of different principal componets (eigenvectors) from PCA for the simulated apo system onto PV (blue line). The eigenvectors in PCA are ordered by the corresponding eigenvalues, which are shown by the red line.

To characterize the experimentally observed piston-like movements triggered by binding of one Asp to the Tar receptor dimer, we first compare the structures of the tar dimer with (holo) and without (apo) Asp bound. Technically, since the only available crystal structure for the *E. coli* periplasmic domain of Tar in the Protein Data Bank (PDB) contains two sulfate ions in each of the binding sites in the dimer and no holo structure of Tar is available for *E. coli*, we modeled the apo and holo structures of the *E. coli* periplasmic domain of Tar based on the two corresponding known structures of Tar in *Salmonella typhimurium* (see Methods for details). Note that these two structures (apo and holo) of Tar in *Salmonella typhimurium* were used previously to support the piston-like model^11^,^13^. From the apo and holo structures of *E. coli* Tar, we calculated the displacements between the two structures for the C_α_ atoms. A piston vector (PV) was then defined as the normalized vector with the displacement components for the C_α_ atoms in the α4 helix only (components of the other C_α_ atoms were set to zero) (see Fig. 3a).

We performed PCA for the biggest conformation cluster of the apo system. The eigenvalues shown in Figure 3b decay rapidly. The projections of PV along each eigenvector in PCA were obtained by the dot product between PV and the eigenvector. As shown in Figure 3b, the first two eigenvectors are dominant based on their eigenvalues. Interestingly, the second dominant PCA eigenvector shows large projection along PV (as large as 0.67), and thus is highly correlated with the piston-like sliding motions of the α4 helix.

**Figure 4:**
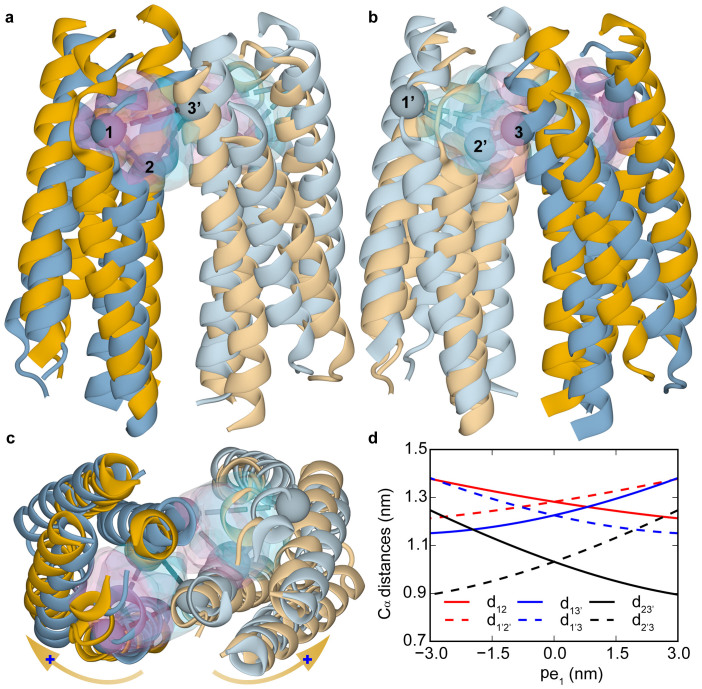
The first principal component from PCA for the apo system. The movement along the first principal component was shown in (a) and (b) for the two opposing (front and back) side views, and (c) for the top view. The two structures, colored blue and orange, correspond to the helices before and after the movement, respectively. The two monomers are distinguished by their different shades of color. This principal component closely corresponds to an overall relative rotation between the two monomers illustrated by the orange arrows with the blue plus sign marking the rotational direction in (c). The two inter-monomer binding pockets were shown as transparent volumes, whose surface color shows its relation to the two structures along the component: pink for the orange colored structure and blue for the blue colored structure. The pairwise distances (dashed lines in (a–c)) among the C_α_ atoms of three key residues (1: Q152, 2: R64 and 3′: R69′ in the other monomer, labeled with spheres in (a–c)) in the pockets were shown in (d). The distances are named as d*_ij_* for the distances between the *i*th and *j*th residues in (d). The solid and dashed lines were for front and back pockets, respectively.

**Figure 5:**
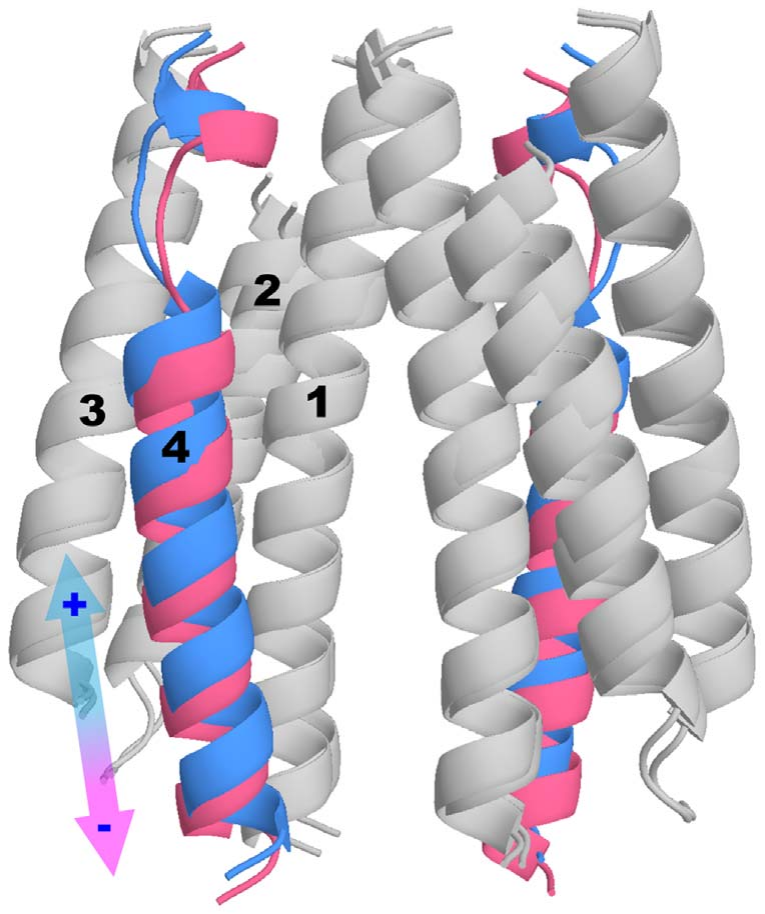
The second principal component from PCA for the apo system. The helices were labeled in one monomer. The direction along the eigenvector was demonstrated by the arrow with both positive and negative direction labels. The negative movement along this principal component contains a downward sliding motion of the α4 helices from blue to pink.

To understand the structure change caused by a given PCA mode, we can move the C_α_ atoms from their average positions in the apo system according to the corresponding PCA eigenvector, and rebuilt the positions of other atoms accordingly. The structure changes induced by the first dominant PCA mode are shown in Figure 4 in different views (Fig. 4a–c). From Figure 4c (the top-view), it is evident that the first PCA mode roughly represents a relative rotation of the two monomers. The most notable structural changes along this eigenvector are the variations in the binding pockets, which are anti-symmetric for the two binding pockets. The pair wise distances among the three key residues (Q152, R64 and R69′) in the binding pockets extrapolated along the first eigenvector were evaluated (see Fig. 4d). When the distance between the two key residues in the front pocket (shown in Fig. 4a for the front-view) increases, the corresponding distance in the back pocket (shown in Fig. 4b for the back-view) decreases, indicating that only one pocket may be available to the ligand. This explains the negative cooperativities between the two binding sites observed in previous experiments^5^. The dominance of this intrinsic rotational motion also indicates the functional relevance of the rotation between the two subunits observed in crystal structure studies^9^,^10^,^11^. We have also studied the structural changes caused by the second dominant PCA mode. As shown in Figure 5, the predominant structural changes correspond to piston-like sliding motions of the two α4 helices in the Tar dimer. This result is consistent with the similarity between PV and the second PCA eigenvector as shown in Figure 3.

To further demonstrate the robustness of the two major modes from PCA, we performed anisotropic network model (ANM) based normal mode analysis (NMA) for the initial structure of the apo system (see Supplementary Fig. S3 for detailed results). The first two normal modes (see Supplementary Fig. S4 for the structure changes along the two normal modes) with the lowest frequencies show high similarities with the eigenvectors in PCA from the MD trajectories. The first normal mode is quasi parallel to the second eigenvector in PCA and PV with high similarities (dot products) of 0.89, and 0.73, respectively. The second normal mode is similar to the first eigenvector in PCA with a similarity of 0.64. We also carried out NMA for the average structure from the apo system simulations. The results (Supplementary Fig. S3b) are quite similar to the NMA results for the initial structure of the apo system. Close correspondences between the normal modes and PCA were also found in other systems^31^. The consistency between the PCA for the full atom MD simulations and the much simpler and highly coarse-grained NMA gave us confidence that the first two eigenvectors in both PCA and NMA capture the intrinsic dynamics of the periplasmic domain of Tar dimer robustly. Additional MD simulations for the periplasmic domain of Tsr receptor dimer also confirms the relevance of these two modes (see Supplementary Fig. S5a).

**Figure 6:**
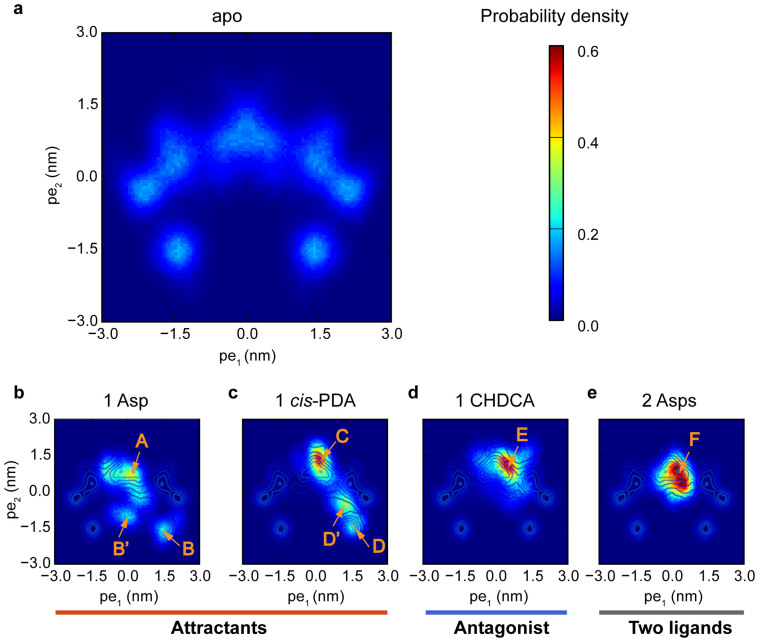
Conformation distributions described by the two dominant principal components. (a) The apo system without ligand. The holo systems bound with (b) 1 Asp, (c) 1 CHDCA, (d) 1 *cis*-PDA, and (e) 2 Asps. Conformations are clustered by the distributions. The clusters are labelled by orange captial letters with arrows. The holo system with 2 Asps is called “Two ligands” to distinguish it from attractants and antagonist.

Having established the relevance of these two principal modes (one rotation mode and one sliding mode), we can now characterize any given Tar dimer structures, e.g., those from the MD simulations, by projecting them onto the two dimensional conformation subspace spanned by these two principal eigenvectors (pe_1_ and pe_2_). As shown in Figure 6a (see also Supplementary Fig. S6a), the apo system has a broad conformation distribution with 5 distinctive conformation clusters, consistent with its broad RMSD and DRMS distributions (Fig. 2). The clusters in the conformation space, as shown in Figure 6a, reflect the intrinsic correlation between the two principal modes. The structures with large negative value of pe_2_ also have large absolute values of pe_1_. In other words, the piston-like motion of the α4 helices are highly correlated with the rotational motions between the two monomers in the apo system. In the following, we report the effects of ligand binding in the four holo systems in the conformation space (pe_1_, pe_2_).

### Single attractant binding stabilizes the conformations with both rotational and piston-like sliding motions

Both Asp and *cis*-PDA are attractants for Tar. The overall characteristics of the two holo systems projected onto the (pe_1_, pe_2_) space were summarized in Supplementary Table S2 (see also Supplementary Fig. S6 for the cluster boundaries). There are three main conformation clusters (A, B′ and B) for the one Asp bound system as shown in Figure 6b. Conformations in cluster A (centered around (pe_1_ = 0.15 nm, pe_2_ = 0.75 nm)) have relatively small values of pe_1_ and pe_2_. Conformations in cluster B (centered around (pe_1_ = 1.53 nm, pe_2_ = −1.83 nm)) have both large positive values of pe_1_, which correspond to significant rotations between the two monomers (see Fig. 4), and large negative displacement projections pe_2_ corresponding to a large downward shift of the α4 helix (see Fig. 5). Similarly, the projection distributions for the 1 *cis*-PDA system (see Fig. 6c) show 3 clusters (labeled with C, D′, and D in Fig. 6c), one of which (D) has large positive displacement projections pe_1_ and large negative displacement projections pe_2_.

We have calculated the probability in each conformation cluster by searching the local probability density peaks and using a recently published clustering method^32^ (see Supplementary Fig. S6 and the Supplementary Methods for details of the clustering method). The total probability for the two clusters in the apo system with large negative values of pe_2_ and large absolute values of pe_1_ is 0.14. In the one Asp bound system, the total probability for the two clusters with negative pe_2_ (B′ and B) is 0.28 (0.13 and 0.15 for B′ and B, respectively). In the 1 *cis*-PDA system, the total probability for the two clusters with negative pe_2_ (D′ and D) is 0.40 (0.25 and 0.15 for D′ and D, respectively) (see Supplementary Table S2). Therefore, the conformations with large negative values of pe_2_ and large absolute values of pe_1_ become more populated (as compared to the apo system) by binding to a single attractant molecule. In other words, both rotational and piston-like sliding motions in the periplasmic domain of Tar dimer were strengthened by the binding of one attractant molecule. Similar behaviors were observed in the Tsr simulations (see Supplementary Fig. S5b).

The ensemble averaged values for pe_1_ and pe_2_ were derived (Supplementary Table S2). Although conformations in B clusters have large negative value of pe_2_ (about −1.5 nm), the ensemble averaged motion for all structures containing A and B conformations is quite limited in the 1 Asp system (Supplementary Table S2, −0.1 nm for Asp). In this way, the ensemble average from the simulations keeps quite close to the downward shift values of α4 helix in the piston-like model^13^,^17^ obtained from the crystal structure comparisons and the downward displacements of simulated kinase-inactive transmembrane helices^18^. Similar behavior is true for pe_1_. In fact, careful comparison^11^,^13^ of the crystal structures have also indicated both a subtle piston-like sliding and a tiny rotations (both small due to the ensemble average effect).

### Antagonist or two attractants binding inhibits both the rotational and the piston-like sliding motions

When we substituted the attractant *cis*-PDA with its antagonist analog CHDCA to bind with the Tar dimer, the conformation clusters with large negative pe_2_ disappeared and only one cluster (E in Fig. 6d) was observed. CHDCA is very similar to *cis*-PDA, only the NH_2_^+^ group in *cis*-PDA was replaced by the CH_2_ group. However, such small change in the ligand molecule has significant effects. It removes the hydrogen bonds with Tar α4 helix, and it also introduces an extra hydrophobic atom to the binding pocket. In particular, the conformations in cluster E with CHDCA bound (Fig. 6d, and Supplementary Fig. S7a for a typical structure) and the conformations in cluster D with *cis*-PDA bound (Fig. 6c, and Supplementary Fig. S7b for a typical structure) have different binding pockets. The main differences are from the relative positions of the residues F150, R69′, and R73′, in the α4, α1′, and α1′ helices, respectively. In the D-like conformations, the F150 residue situates between R69′ and R73′, while it flanks on top of R73′ in the E-like conformations. The steric effects of F150 make large ligands difficult (especially for hydrophobic molecules with the same hydrophobic properties as F150) to bind to the receptor in D-like conformations. This explains why only cluster E was observed in the CHDCA bound system (see Supplementary Fig. S7c for a typical complex structure with E conformations). For *cis*-PDA, the extra hydrogen bonds and favored electrostatic potential distributions can turn F150 to the top of R73′, making binding to D-like conformations (see Supplementary Fig. S7d for the typical complex structure with D conformations) possible. These results demonstrate the importance of the amino groups of the ligands and the hydrogen bonds with the α4 helix in stabilizing the signaling conformations.

For the system with two Asp bound, the conformation also has only one cluster as shown in Figure 6e. Due to the anti-symmetric properties of the first principal mode pe_1_, the two Asp bound system prefer the conformations with pe_1_ near zero. Large positive or negative values of pe_1_ would be incompatible with the bindings of two identical ligands simultaneously to the two anti-symmetric binding pockets (see Fig. 4). Due to the coupling between the two modes, for systems with small values of pe_1_, pe_2_ is also restricted to be near zero, which eliminates the downward sliding of the α4 helix. As shown in Figure 6b, the one Asp bound system has three clusters, A, B and B′. The A-like conformations have two available binding pockets (see Supplementary Fig. S8a and S8b) with similar sizes. However, the two pockets are smaller in B-like conformations (Supplementary Fig. S8c and S8d). Thus, B-like conformations only allows one ligand binding (Supplementary Fig. S8e and S8f). The B′-like conformations is quite rare in the apo system and the two pockets for B′-like conformations fall in between the A and B like conformations in size (Supplementary Fig. S8g and S8h). With one Asp bound in one pocket (Supplementary Fig. S8g), conformation B′ also shrank the other vacant pocket dramatically (Supplementary Fig. S8h). Therefore, the two Asps bound system would only stabilize and adapt the A-like conformations (F cluster for 2 Asps, see Fig. 6e). Note that the A-like conformations (A, C, E, and F in Fig. 6) also have negative cooperativity between the two binding pockets due to the anti-symmetric effects of pe_1_. This is even true for the antagonist (CHDCA), which prefer to bind to one of the pockets in E-like conformations (see Supplementary Fig. S9). Our results thus suggest that two attractants binding, which can only occur at high ligand concentrations, can disrupt or even eliminate the attractant response by forcing the receptor dimer into a symmetric non-signaling state. In fact, Yeh *et al.*^9^ have solved the crystal structure with two aspartate bound by soaking in 42 mM Asp solution. The resulting complex structure indeed did not show any piston-like motion compared with the apo structures^9^.

### The relative contributions of the α4 helix to ligand binding free energy

To understand the energetics of ligand binding, we measured the fraction of hydrogen bonding contacts (see Supplementary Fig. S10) between ligands and the key atoms in the receptor binding pocket. All ligands form strong hydrogen bonds with residues R69′, R73′ and R64 of the Tar dimer. In the 1 Asp, 1 *cis*-PDA and 2 Asps systems, ligands form hydrogen bonds dynamically with the main-chain carbonyls of Y149, F150, Q152 in the α4 helix and the side chain oxygen atoms of T154. However, these hydrogen bonds are absent in the 1 CHDCA system due to lack of NH_3_^+^ or NH_2_^+^ group in CHDCA.

The binding free energies and the α4 helix contributions were evaluated for the representative conformations (Table S3). Though the total binding free energies do not vary widely, the relative contributions from the α4 helix do differ significantly. For the 1 Asp, 1 *cis*-PDA and 2 Asps systems, the interactions between the ligands and the key atoms in α4 helix contribute over −13 kcal**·**mol^−1^ to the total ligand binding free energy. In contrast, CHDCA shows much weaker interactions with the α4 helix, only contributing −4.5 kcal**·**mol^−1^ to an otherwise similar total ligand binding free energy. Although there are strong α4 helix interactions in the 2 Asps system, the symmetry constraint prevents the system from having large pe_1_ conformations as we described before. Overall, our results suggest that the interactions between the amino group of the ligands and main chain carbonyl oxygen atoms of Y149 and Q152 as well as the side chain oxygen atom of T154 in the α4 helix help stabilize the signaling conformations (B, B′, D, D′ in Fig. 6) provided these conformations are allowed by symmetry. These results are compatible with the fractions of hydrogen bond contacts (see Supplementary Fig. S10).

## Discussion

In this paper, we have used MD simulations to study the conformation dynamics of the apo system and the conformation changes in the holo systems for *E. coli* Tar periplasmic domain. PCA was used to analyze the conformation dynamics obtained from MD simulations. We found that the conformation dynamics can be largely represented by two dominant principal components (modes). The first principal component corresponds to a relative rotation of the two monomers in the receptor dimer, likely due to the electrostatic repulsive interactions between the two monomers at the dimer interface^21^. This relative rotational movement provides a flexible pocket size for binding to a diverse set of ligands. Furthermore, this anti-symmetric movement results in a negative cooperativity between the two binding pockets, which have been considered as a general mechanism for signal transductions^33^. The second principal component corresponds to a (piston-like) sliding motion of the α4 helix relative to the other helices, which is consistent with the piston-like model. These two principal modes are highly correlated. We found that a single attractant (1 Asp or 1 *cis*-PDA) binding to the Tar dimer stabilized the conformations with both large rotational motion (pe_1_) and large downward sliding of the α4 helix (pe_2_). On the other hand, binding of the antagonist molecule CHDCA, or two Asps simultaneously, suppresses both modes. Crystal structures obtained under different conditions^4^,^6^,^7^,^8^,^9^,^10^,^11^ confirmed the existence and functional relevance of the two modes. Our results suggest a mechanism of signaling in bacterial chemoreceptor dimers that involves both inter-monomer rotation and piston-like sliding of the α4 helix in the periplasmic domains of the chemoreceptors.

Hall *et al.*^18^ and Park *et al.*^19^ have also used MD simulations to study the signaling related structure changes of chemoreceptors. They focused on structure changes of the transmembrane and HAMP domains rather than the periplasmic domain. Hall *et al.*^18^ found the piston-like motions in the transmembrane domain. In the simulation studies by Park *et al.*^19^, the structures with piston-like downward sliding in the periplasmic domain were enforced by restraints during simulations. They observed changes in both the structure and the flexibility of the receptor in the transmembrane and HAMP domains. In principle, the two kinds of motions in the periplasmic domain shown in our study could induce quite complex changes in the downstream domains, including rotational and/or piston-like motions in the transmembrane^18^,^19^ and HAMP domains^19^,^34^,^35^, the bending motions in the glycine hinge regions^36^, the conformation changes of the residues near the cytoplasmic tips^30^, and even more complex structure changes in higher clustering levels of oligomers^37^. It would be interesting to develop a full length receptor model to study how receptor conformational changes in transmembrane and HAMP domains are triggered directly by ligand binding. Although there were simulations for the periplasmic domain, transmembrane domain^18^,^19^, HAMP domain^19^, cytoplasmic domain^30^, and even trimers-of-dimers^38^ separately, no reported simulation considers the full length chemoreceptors and CheA/CheW complexes. It is unclear how signals transmit from the binding pockets in the periplasmic domain of the receptor to the downstream kinase CheA far from the periplasmic domain step by step. As the structural information of the individual proteins in the complex and their interactions become available^30^,^39^,^40^,^41^, a residue level coarse-grained models^38^,^42^ may be developed to help elucidate the molecular mechanisms of signaling in bacterial chemoreceptors.

## Methods

### MD simulations

We performed explicit solvent MD simulations for five systems with the periplasmic domain of Tar dimer (residues: 36–181) in *E. coli*, including the apo system, holo systems containing Tar dimer bound with 1 Asp, 1 CHDCA, 1 *cis*-PDA and 2 Asps, respectively. The initial configurations were prepared using similar protocols as reported in our previous study^25^ except for the structure templates of receptors. As the only available crystal structure for the *E. coli* periplasmic domain of Tar in PDB contains two sulfate ions in each of the binding sites in the dimer and no holo structure is available, we first modeled the apo and holo structures of the *E. coli* periplasmic domain of Tar based on a pair of crystal structures^4^ (PDB code: 1LIH and 2LIG for apo Tar and Tar bound with an Asp respectively) for Tar periplasmic domain of *Salmonella typhimurium*. The foreign molecules in the PDB files (phenanthroline, sulfate ions and water molecules) were deleted and the sequences were mutated to the sequence of *E. coli* receptor. The initial configuration for ligand Asp was taken directly from the crystal structure (PDB code: 2LIG). Other initial configurations for the holo systems were obtained using similar docking method as in our previous study^25^. We also simulated two systems with the periplasmic domain of Tsr dimer (residues: 36–183) in *E. coli*, including the apo system and the holo system containing Tsr dimer bound with one Ser. The simulation details were similar but with shorter simulation time (5 independent 200 ns runs for each system). The initial structures were from the apo crystal structure^43^ (PDB code: 2D4U) for Tsr in *E. coli*.

The systems were described by AMBER ff99SB force field^44^ for proteins, combined with TIP3P model^45^ for water molecules, and, the general AMBER force field (GAFF)^46^ for ligands.

All simulations were performed with GROMACS version 4.5.4^47^. The systems were placed in a dodecahedral water box with at least 1.5 nm of solvent on all sides. Sodium or chlorine ions were added to produce a neutral charge system. Periodic boundary conditions (PBC) were used. Cutoffs for both van der Waals and electrostatic interactions were 0.9 nm. Long range electrostatic interactions were treated by the particle mesh Ewald (PME) method. The simulations were performed in the NPT ensemble at 300 K and 1 bar. We used Langevin dynamics with a friction constant 0.5 ps^−1^ as a thermostat, and Berendsen pressure coupling method with a time constant 2.0 ps and a system compressibility 4.5 × 10^−5^ bar^−1^ to control system pressure. The equations of motion were integrated with a leap-frog algorithm^48^ with a time step of 2 fs.

Minimization and equilibration were done, followed by 5 independent 500 ns production simulations for Tar systems and 5 independent 200 ns simulations for Tsr systems. Production simulations were run in NPT ensembles with different seeds for the random number generator. In all simulations, the first 20 ns trajectories were not used for analysis to ensure proper equilibration, and the other trajectories for the same systems were combined together for further analyses. Swapping the symmetric chains extended the trajectories and enhanced the statistics for the apo system.

### PCA for simulated trajectories

To understand the structure dynamics, we carried out PCA to study protein collective dynamics^31^. The covariance matrix was calculated from MD trajectories. The eigenvectors in the order of the magnitude of corresponding eigenvalues for principal components were obtained by diagonalization of the covariance matrix in apo system. The first two eigenvectors are dominant. The displacements from the average structure of the apo system to a given snapshot could then be projected onto the two dominant eigenvectors to measure structure deformations along the corresponding principal component directions. The trajectories for the apo system and the holo systems with different ligands were analyzed based on this technique. We also carried out PCA based on the covariance matrix for the holo systems with ligands. The first several eigenvectors for holo systems were also dominant and mainly in the subspace spanned by the first two eigenvectors of the apo system.

### ANM based NMA for the structures in the apo system

We used the ANM based NMA^31^ to further examine the system dynamics for the apo system. In ANM, residue pairs within a cutoff distance R_c_ in a predefined structure were connected by springs with spring constants γ. The eigenvectors (also called the normal modes) and the corresponding frequencies were then obtained from the symmetric eigen problems of the Hessian matrix. Many forms of γ were developed to get better correspondences with the protein dynamics measured in experiments^31^,^49^,^50^. Different choice of the uniform spring constant γ would not change the directions of the eigenvectors.

Here we used a python package ProDy^51^ to get the normal modes based on ANM with default parameters, i.e., R_c_ = 1.5 nm and an uniform γ = 100 kcal**·**mol^−1^**·**nm^−2^, for several apo structures.

### MM/GBSA binding energy prediction

We used the MM/GBSA method (based on the modified GB model developed by Onufriev *et al.*^52^) to predict the binding free energies in the holo systems. The salt concentration was set 0.1 M. The trajectory displacement projections along the principal components were then split into small pieces on grids for the holo systems. The binding free energies could be calculated for all sub-ensembles of structures on grids in principle.

According to our previous study^25^, the main-chain carbonyls of Y149 and/or Q152 with amino or hydroxyl groups in attractants are critical in triggering downstream signaling. To test these specific interactions in holo systems, we used the residue based free energy decomposition method^53^ in AMBER to calculate the binding free energy contributions.

## Author Contributions

Y.T. and L.L. conceived this project. D.Y., Y.T. and L.L. designed the simulations. D.Y. and X.M. performed the simulations. All authors analyzed the data and wrote the manuscript.

## Supplementary Material

Supplementary Information Supplementary Information
