# Supplementary material for: Both piston-like and rotational motions are present in bacterial chemoreceptor signaling
Source: Sci Rep. 2015 Mar 2;5:8640. doi: 10.1038/srep08640 (PMC4345343; doi:10.1038/srep08640)
Supplement: Supplementary Information [file srep08640-s1.pdf]

Supplementary Information

# Both piston-like and rotational motions are present in bacterial chemoreceptor signaling

Daqi Yu[12#], Xiaomin Ma[2#], Yuhai Tu[23*], and Luhua Lai[124*]

[1] BNLMS, State Key Laboratory for Structural Chemistry of Unstable and Stable Species, College of Chemistry and Molecular Engineering, Peking University, Beijing 100871, China.

[2] Center for Quantitative Biology, Academy for Advanced Interdisciplinary Studies, Peking University, Beijing 100871, China.

[3] IBM T. J. Watson Research Center, Yorktown Heights, New York 10598, USA.

[4] Peking-Tsinghua Center for Life Sciences, Peking University, Beijing 100871, China.

[*] Corresponding authors:

Luhua Lai

College of Chemistry and Molecular Engineering, Peking University

Beijing 100871, China.

E-mail: lhlai@pku.edu.cn; Tel: 86-10-62757486

Yuhai Tu

IBM T. J. Watson Research Center, Yorktown Heights

New York 10598, USA

E-mail: yuhai@us.ibm.com; Tel: (914)945-2762

#: These authors contributed equally to this work.

Supplementary Figures S1-S10, Supplementary Tables S1-S3, Supplementary Methods, and Supplementary References.

Supplementary Figures

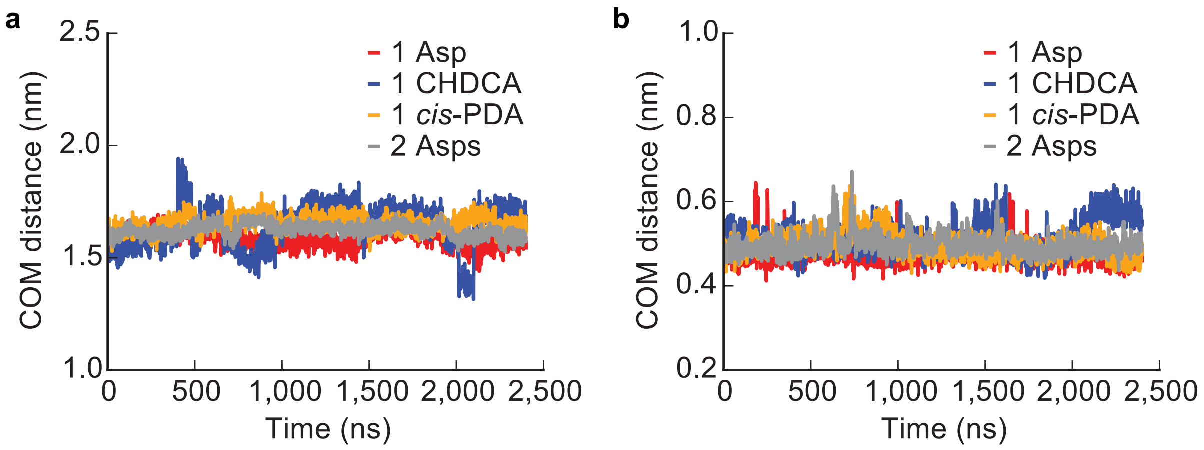

Figure S1: Time serials for the distances between ligands and Tar. Distances between the center of masses (COM) for ligands and (**a**) receptor or (**b**) R69' (awell-known key residue for binding) in the binding pockets were shown with simulation times. In the systems with 2 Asps, the corresponding distances for the 2 Asps were averaged.

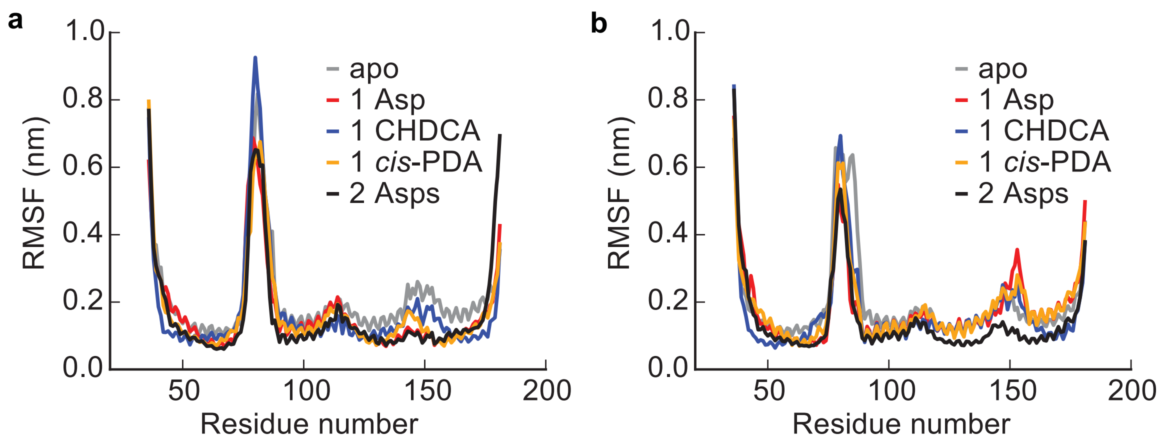

Figure S2: RMSF of Tar dimer in the simulated systems. RMSF for the A and B monomers of Tar dimer in the simulated systems were shown in panels (**a**) and (**b**) respectively. Residues are numbered conventionally according to their apperances in the full amino acids of Tar in *E. coli* in UniProt.

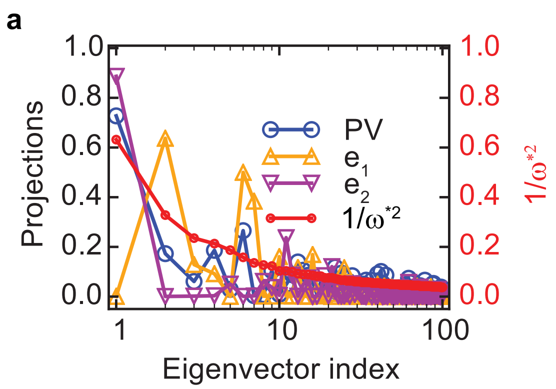

Figure S3: ANM based normal mode analysis for the apo system. The structures for ANM model was based on (**a**) the crystal structure (PDB code: 1LIH) and (**b**) the averaged structures in MD trajectories in the apo system, respectively. The projections of PV and the first two eigenvectors in PCA (e_1, e_2) on the eigenvectors were shown with the left vertical axis. The reciprocals of the reduced square intrinsic mode frequencies comparable to the eigenvalues in PCA were shown with the right vertical axis. The reciprocals were divided by the sum for all modes to get the normalized values.

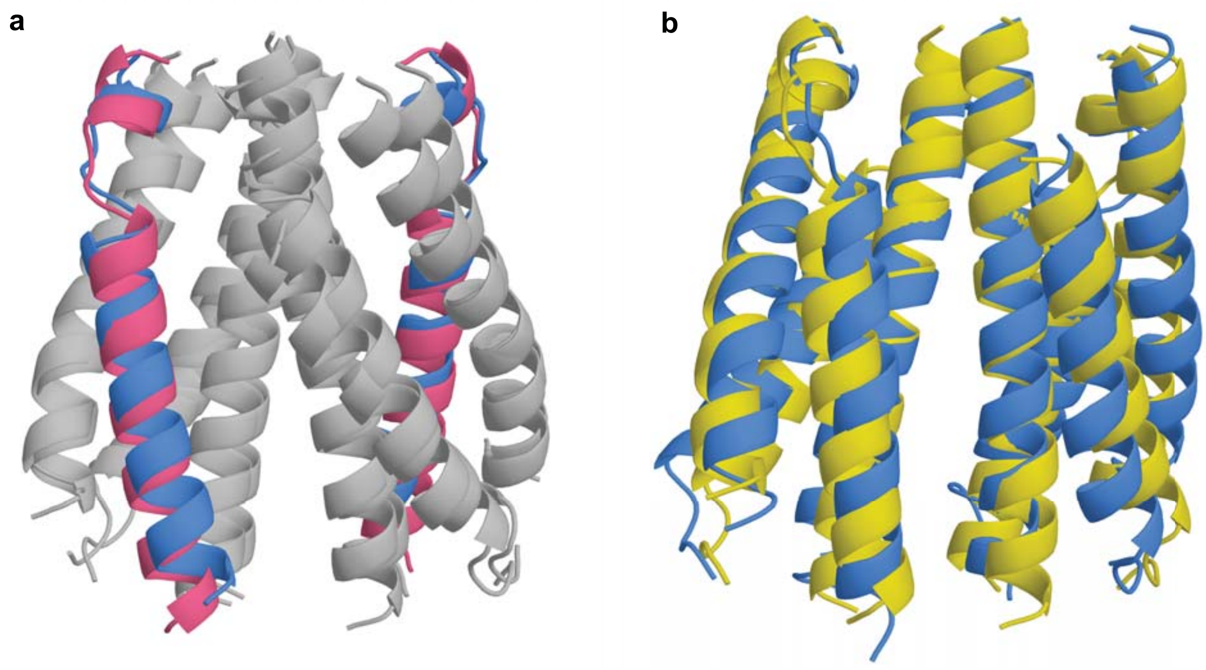

Figure S4: The structure changes along the first two normal modes. (**a**) and (**b**) are for the first and second normal modes based on the crystal structures (PDB code: 1LIH).

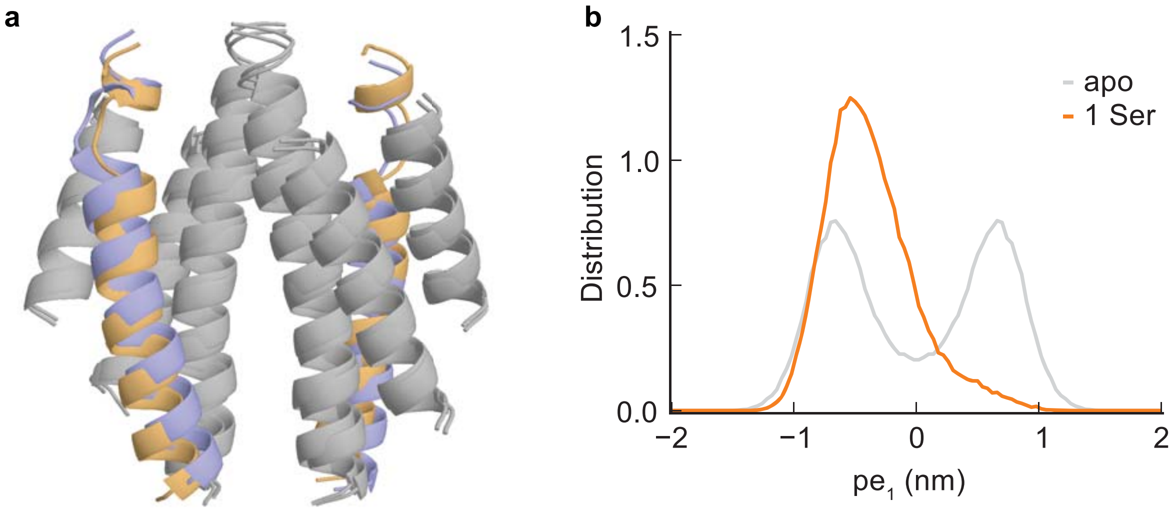

Figure S5: The first principal component in the Tsr simulation systems. (**a**) The movement of the apo system within the first principal component were illustrated by the structure extrapolations along the eigenvector. (**b**) The projection distributions for the simulated trajectories along the eigenvector pe_1 in the apo and holo systems.

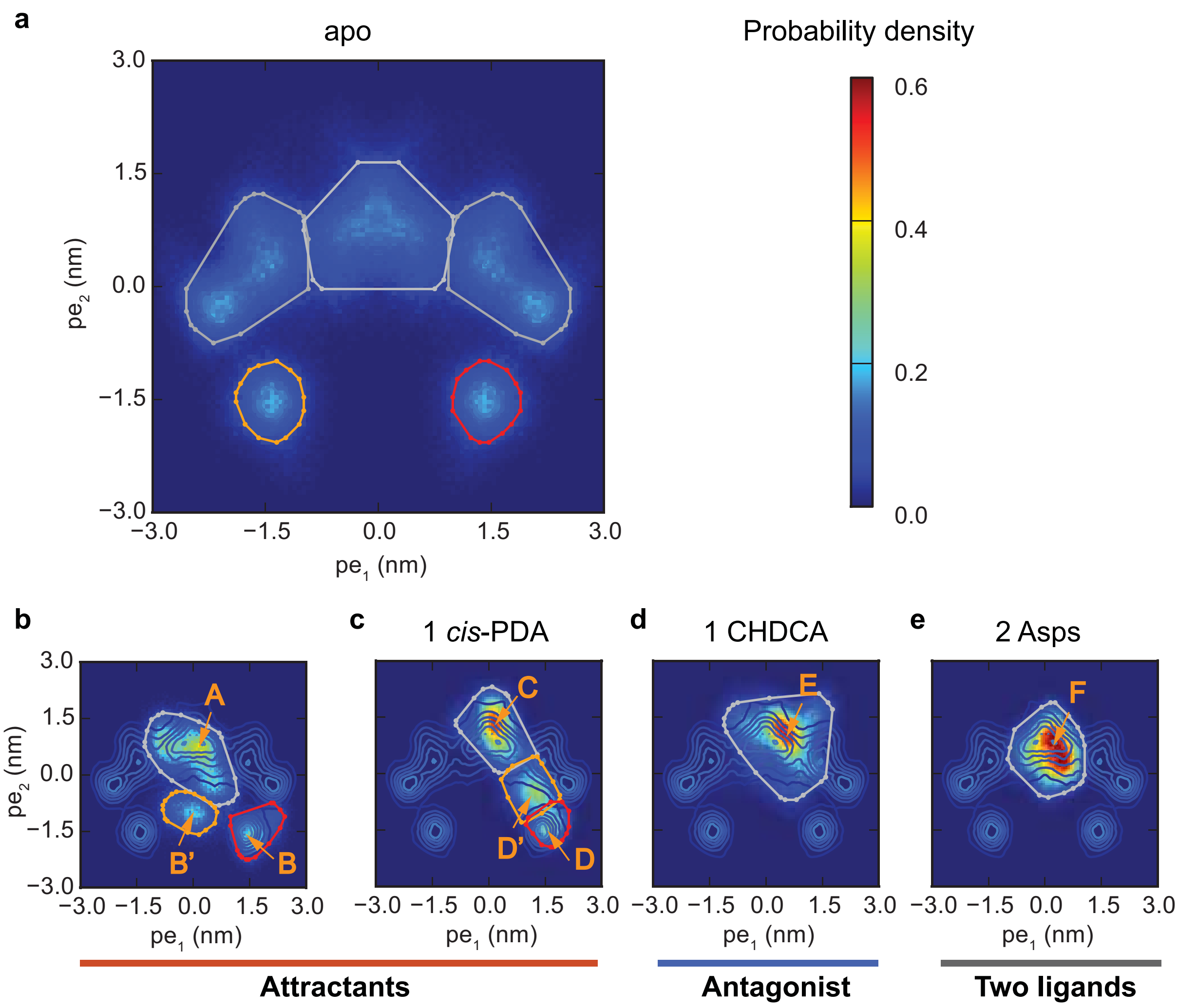

Figure S6: Conformation clusters described by the two dominant principal components. The figure is an extension to Figure 6 in the main text. (**a**) The apo system without ligand. The holo systems bound with (**b**) 1 Asp, (**c**) 1 CHDCA, (**d**) 1 *cis*-PDA , and (**e**) 2 Asps. Conformations are clustered by the distributions. The clusters are labelled by orange captial letters with arrows. We call the holo system with 2 Asps "Two ligands" here to indicate the symmetric binding effect that is irrelevant to the taxis functions (attractant, antagonist, or repellent) of two ligands. The conformation clusters were counted by the method described in the Supplementary Methods part. The boundary of all clusters are illustrated by the convex hull containing the grids in the cluster using different colored lines with dots.

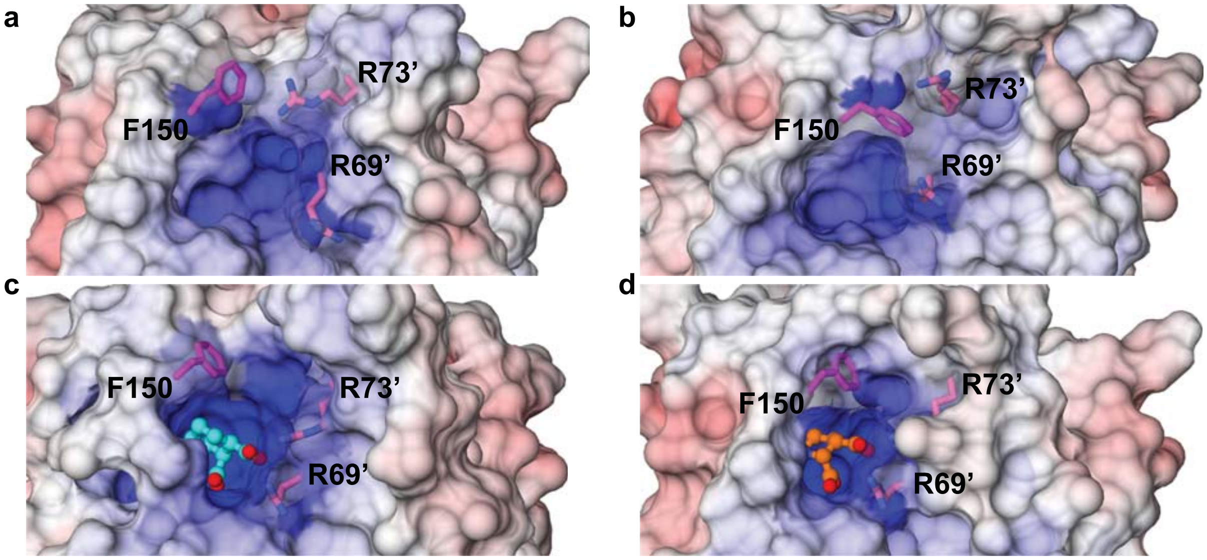

Figure S7: Different conformations stabilized by different properties of CHDCA and *cis*-PDA. The MSMS molecular surface was shown in Chimera[1] with the electrostatic potential calculated by APBS package[2] mapped colors. To show the binding pockets, the three key residues (F150, R69’ and R73’), and the ligands (if exists) simultaneously, we set the surface near the three key resides and ligands transparent. Three key residues in the binding pockets were shown with sticks colored as magenta colors and the ligands were shown as ball and sticks. (**a**) and (**b**) are the typical structures for clusters in the apo system that are homologous to the E and D conformations respectively. (**c**) is a typical structure for E conformations in 1 CHDCA system, and (**d**) is a typical structure for D conformations in the 1 *cis*-PDA system..

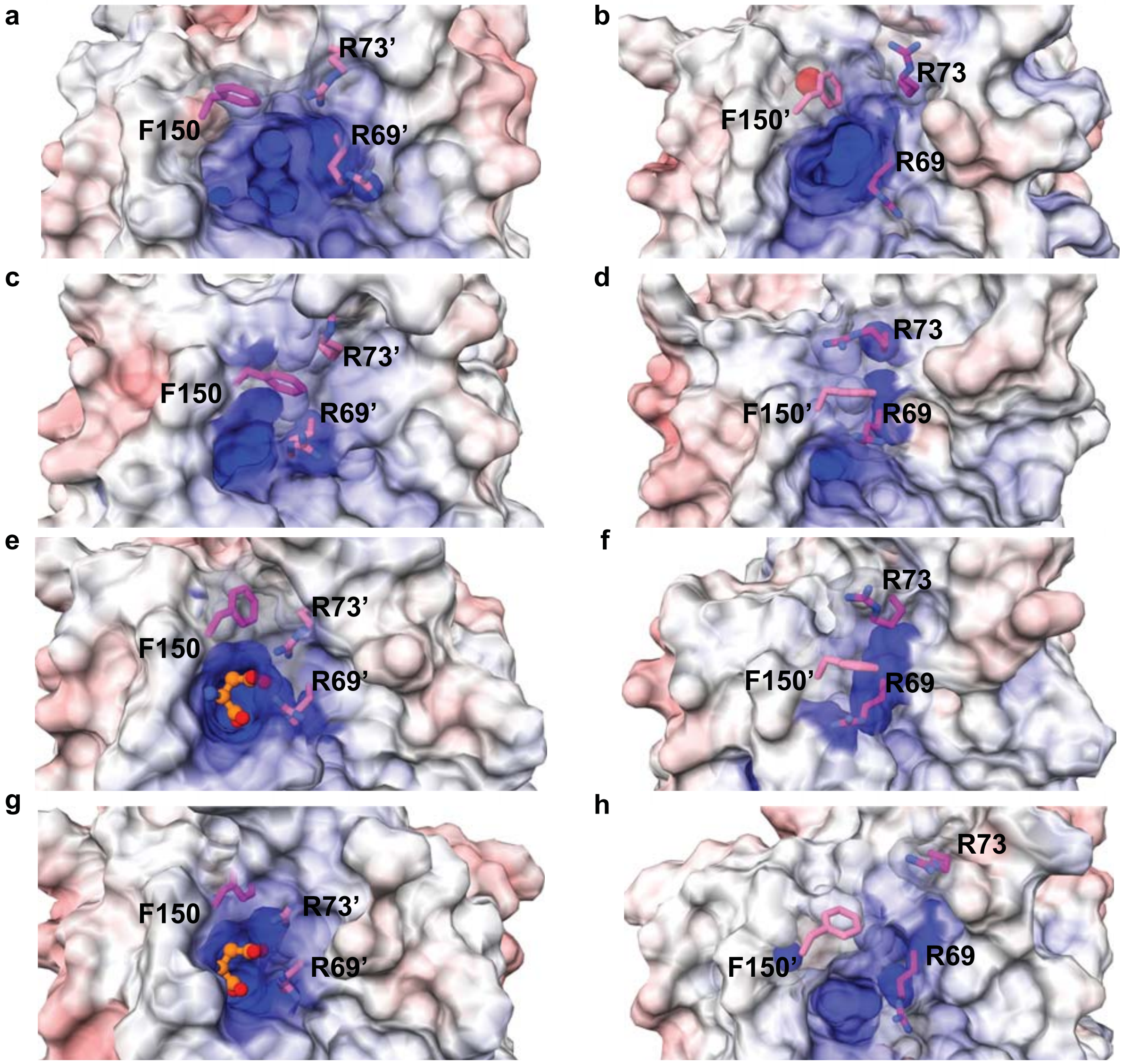

Figure S8: Pockets for the conformations in the 1 Asp system. The surface and colors were the same as in Supplementary Figure. S7. (**a**) and (**b**) are the front and back views for the typical structure with A-like conformations in the apo system. (**c**) and (**d**) are the front and back views for the typical structure with B-like conformations in the apo system. (**e**) and (**f**) are the front and back views for the typical structure with B conformations in the 1 Asp system. (**g**) and (**h**) are the front and back views for the typical structure with B' conformations in the 1 Asp system.

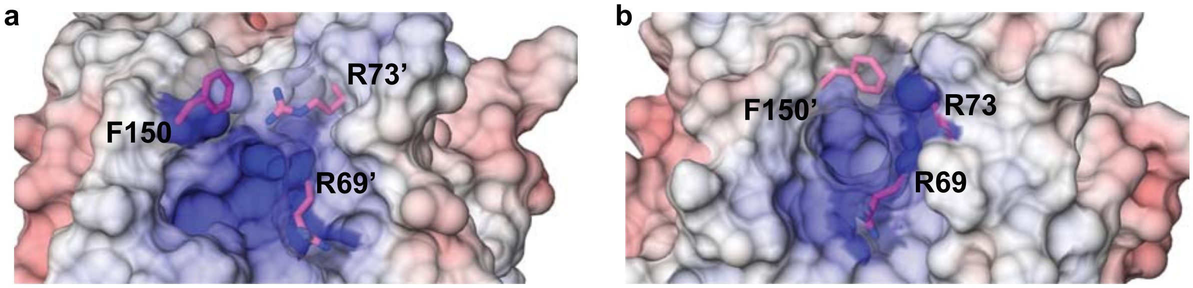

Figure S9: Pockets for the 1 CHDCA stabilized conformation. The surface and colors were the same as in Supplementary Figure S7. (**a**) and (**b**) are the back views for the typical structures in E-like conformations in the apo system and the 1 CHDCA system bound with a CHDCA in the front pocket. They are the back views for Supplementary Figure S7a and S7c respectively.

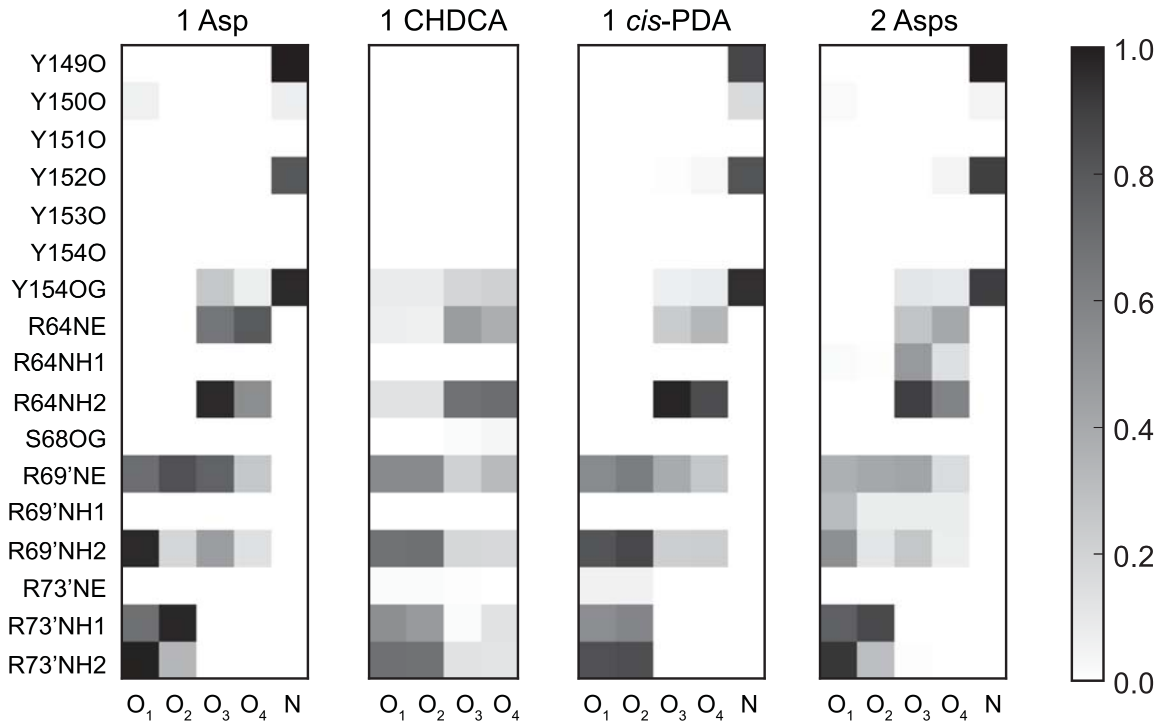

Figure S10: Fractions of contact maps for hydrogen bonds between ligands and Tar. The atoms of ligands were labelled in the bottom and the atoms in the receptor binding pockets were shown in the left. In the 2 Asps system, the corresponding distances for the 2 Asps were averaged.

Supplementary Tables

Table S1. The simulation systems

#	Template	Ligand	Function	Atoms	Name
1	1LIH	-	-	83,308	apo
2	2LIG	1 Asp	Attractant	80,445	1 Asp
3	2LIG	1 CHDCA	Antagonist	80,457	1 CHDCA
4	2LIG	1 *cis*-PDA	Attractant	80,443	1 *cis*-PDA
5	2LIG	2 Asp	ND[a]	80,456	2 Asps

[a] No direct experiment measurements in ultrahigh concentrations.

Table S2. The projection characteristics on the first two principal components

System	# of Clusters	Cluster Labels	Density Peak (nm)	Probability[b]	Ensemble Average (nm)
apo	5	NL[a]	(2.19, -0.39)	0.20	(0, 0)
			(-2.19, -0.39)	0.20	
			(0.27, 0.81)	0.23	
			(1.41, -1.47)	0.07	
			(-1.41, -1.53)	0.07	
1 Asp	3	A	(0.15, 0.75)	0.60	(0.25, -0.10)
		B'	(-0.03, -0.93)	0.13	
		B	(1.53, -1.83)	0.15	
1 *cis*-PDA	3	C	(0.15, 1.17)	0.52	(0.73, 0.26)
		D'	(1.29, -0.57)	0.25	
		D	(1.65, -1.41)	0.15	
1 CHDCA	1	E	(0.63, 1.05)	0.89	(0.44, 0.93)
2 Asps	1	F	(0.39, 0.33)	0.95	(0.17, 0.59)

a The clusters were not labelled in the apo system. b The results were obtained from the projection distributions (see Supplementary Fig. S6 for the cluster boundaries and Supplementary Methods for the counting details).

Table S3. Ligand-receptor binding energies in the holo systems

System	Clusters	Δ G (kcal•mol^{-1})	ΔG$_I$[a] (kcal•mol^{-1})
1 Asp	A	-54.4±4.0	-14.6±1.4
	B'	-55.3±3.6	-14.5±1.3
	B	-50.4±4.1	-14.7±1.5
1 *cis*-PDA	C	-56.8±4.6	-13.3±1.2
	D'	-56.9±4.0	-13.3±1.1
	D	-54.5±4.1	-13.4±1.1
1 CHDCA	E	-51.0±8.5	-4.5±3.8
2 Asps	F	-50.9±3.3	-15.8±1.3

[a] Free energy contributions by the interactions between the ligand and the α4 main chain atoms of Y149, F150, Q152, and the side chain atoms of T154.

Supplementary Methods

Calculating the probabilities for the projection clusters

The clusters in Supplementary Table S2 were obtained by searching the local density peaks of the projection distributions shown in Figure 6. We have noted that Rodriguez and Laio[3] have developed an automatic and robust clustering method based on searching local density peaks. We thus used an adapted method to count the cluster probabilities. Since the number of the structure coordinates in our trajectories is quite large, we used the distributions of the trajectory displacement projections along the first two principal components (Fig. 6) as the clustering objects. That is to say, we used the densities on the grids, searched the local density peaks, and assigned a cluster number to each grid according to the method developed by Rodriguez and Laio[3]. The advantage of this protocol is that it gives qualitative results and keeps the positions of the density peaks equal to those manually picked. In practice, we dropped the grids with quite small densities (less than 0.05 nm^{-2} here) to further accelerate the calculations and sharpen the clusters with clearer boundaries. Once the clusters were determined, the cluster probabilities were calculated by integrate the densities on the grids.

Supplementary References

1 Pettersen, E. F. *et al.* UCSF Chimera--a visualization system for exploratory research and analysis. *J. Comput. Chem.* **25**, 1605-1612, doi:10.1002/jcc.20084 (2004).

2 Baker, N. A., Sept, D., Joseph, S., Holst, M. J. & McCammon, J. A. Electrostatics of nanosystems: application to microtubules and the ribosome. *Proc. Natl. Acad. Sci. U. S. A.* **98**, 10037-10041, doi:10.1073/pnas.181342398 (2001).

3 Rodriguez, A. & Laio, A. Clustering by fast search and find of density peaks. *Science* **344**, 1492-1496, doi:10.1126/science.1242072 (2014).
